# Recombinant Expression of Thrombolytic Agent Reteplase in Marine Microalga *Tetraselmis subcordiformis* (Chlorodendrales, Chlorophyta)

**DOI:** 10.3390/md19060315

**Published:** 2021-05-28

**Authors:** Chunhui Wu, Caiyun Zheng, Jinxia Wang, Peng Jiang

**Affiliations:** 1 CAS and Shandong Province Key Laboratory of Experimental Marine Biology, Center for Ocean Mega-Science, Institute of Oceanology, Chinese Academy of Sciences, Qingdao 266071, China; wuchunhui@qdio.ac.cn (C.W.); caiyun.zheng140205@mail.nwpu.edu.cn (C.Z.); jxwang@qdio.ac.cn (J.W.); 2 Laboratory for Marine Biology and Biotechnology, Qingdao National Laboratory for Marine Science and Technology, Qingdao 266237, China

**Keywords:** algal genetic transformation, recombinant expression, Reteplase, *rt-PA* gene, *Tetraselmis subcordiformis*, thrombolytic agent

## Abstract

*Tetraselmis subcordiformis*, a unicellular marine green alga, is used widely in aquaculture as an initial feeding for fish, bivalve mollusks, penaeid shrimp larvae, and rotifers because of its rich content of amino acids and fatty acids. A stable nuclear transformation system using the herbicide phosphinothricin (PPT) as a selective reagent was established previously. In this research, the recombinant expression in *T. subcordiformis* was investigated by particle bombardment with the *rt-PA* gene that encodes the recombinant human tissue-type plasminogen activator (Reteplase), which is a thrombolytic agent for acute myocardial infarction treatment. Transgenic algal strains were selected by their resistance to PPT, and expression of *rt-PA* was validated by PCR, Southern blotting, and Western blotting, and bioactivity of rt-PA was confirmed by the fibrin agarose plate assay for bioactivity. The results showed that *rt-PA* was integrated into the genome of *T. subcordiformis*, and the expression product was bioactive, indicating proper post-transcriptional modification of rt-PA in *T. subcordiformis*. This report contributes to efforts that take advantage of marine microalgae as cell factories to prepare recombinant drugs and in establishing a characteristic pathway of oral administration in aquaculture.

## 1. Introduction

*Tetraselmis subcordiformis* (Wille) Butcher (= *Platymonas subcordiformis*) is a common marine unicellular green alga with broad application prospects in aquaculture and bioenergy [1,2,3,4]. Due to its high quantities of polysaccharides, proteins, polyunsaturated fatty acids, vitamins, and other nutrient supplements [4,5,6,7], *T*. *subcordiformis* is widely used in aquaculture as feed for bivalve mollusks, penaeid shrimp larvae, and rotifers [2,8,9]. In addition, *T*. *subcordiformis* was shown to photobiologically evolve hydrogen (H_2_), indicating that this alga may also provide a potential sustainable energy source [3].

In earlier research, both stable nuclear and chloroplast transformation systems have been established in *T. subcordiformis* using glass-bead agitation and the particle bombardment method [10,11]. Among three proven effective exogenous promoters, the transformation rate of the *egfp* gene in *T. subcordiformis* driven by CaMV35S and SV40 promoters was higher than that of the CMV promoter [12]. GFP as a reporter gene was successfully expressed in the cytoplasm and chloroplast of *T. subcordiformis* to display the success of transformation [11,12,13]. Stable inheritance of a foreign character can be obtained by screening with a resistance gene. *T. subcordiformis* has been shown to have no sensitivity to streptomycin, kanamycin, spectinomycin, or chloramphenicol, but is highly sensitive to the herbicide phosphinothricin (PPT), whose brand name is Basta [10]. The Basta resistance gene *bar* confers PPT resistance to positive transformants of *T. subcordiformis* [10]. Therefore, developing *T*. *subcordiformis* as a eukaryotic, photoautotrophic, and low-cost cell factory to prepare high value bioactive substances such as recombinant drugs is a promising approach.

Reteplase (rt-PA) is the third generation of thrombolytic agents for treating acute myocardial infarction and structurally a single-chain non-glycosylated deletion mutant of wild-type tissue plasminogen activator (t-PA), consisting of only the Kringle II and protease domain [14,15]. The affinity of rt-PA toward fibrin is greater than 600-fold higher when compared with that of t-PA. Moreover, rt-PA fails to bind with human hepatocellular specific receptors because of the absence of Kringle I and the EGF domain, which prolongs the half-life of rt-PA in blood [16,17,18]. Although rt-PA is the most effective agent to treat clinical thrombolysis, high preparation costs from incorrect protein folding and disulfide bond modifications in prokaryotic expression systems make preparation of this agent expensive, thereby precluding easy access, especially in developing countries. To reduce the preparation costs, in this study, heterologous expression of the *rt-PA* gene was investigated in a eukaryotic microalga *T*. *subcordiformis*, with bioactivity of the produced recombinant rt-PA evaluated. In addition, the potential of transgenic *T*. *subcordiformis* as a new marine bioreactor to produce recombinant drugs and as a characteristic pathway for oral administration in aquaculture is discussed.

## 2. Results

### 2.1. Biolistic Transformation and Basta Selection

**Figure 1:**
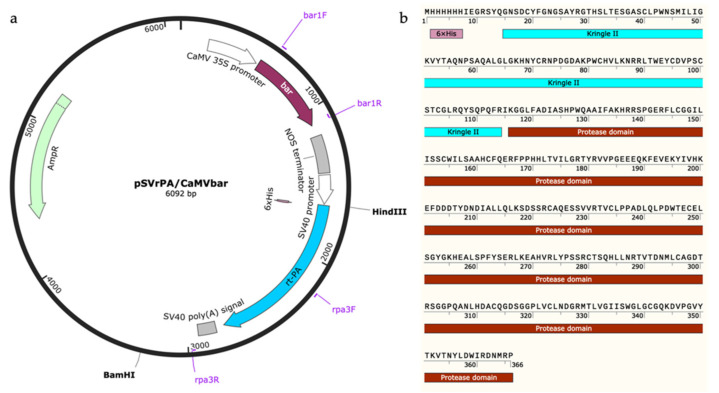
Illustration of plasmid vector pSVrPA/CaMVbar used to transform *T*. *subcordiformis* (**a**) and representation of the rt-PA protein which contains His-tag (pink), Kringle II domain (blue-green), and Protease domain (brown) (**b**).

The heterologous protein expression vector pSVrPA/CaMVbar constructed in our laboratory was used in this experiment. The herbicide PPT resistance gene *bar* was driven by the CaMV35S promoter (Figure 1a). The rt-PA protein consists of three modules, namely, integrated His-tag, the Kringle II domain, and the protease domain (Figure 1b), and expression of the *rt-PA* gene was regulated by the SV40 promoter. Genetic transformation was performed using the particle bombardment method.

**Figure 2:**
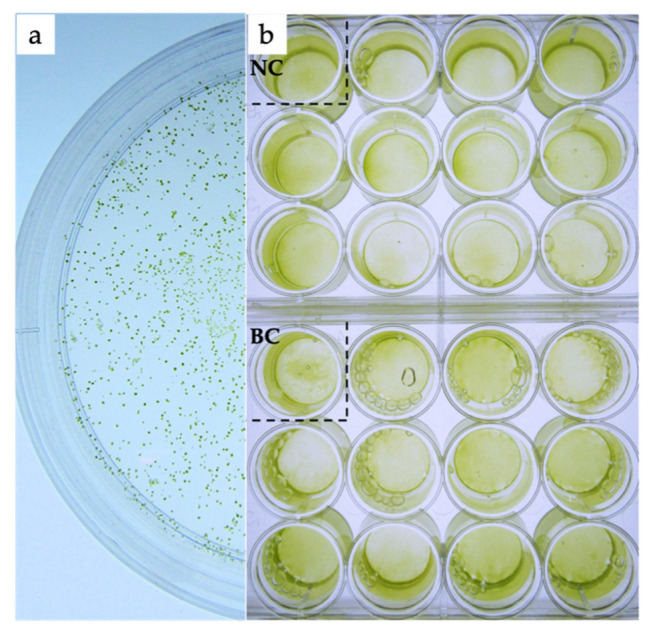
Herbicide PPT screening of transformed *T. subcordiformis*. (**a**) The PPT-resistance colonies appeared in solid modified f/2 medium containing 40 mg·L^−1^ of PPT; (**b**) culture of wildtype group, the negative control, and 22 PPT-resistance colonies without herbicide. BC, blank control, wildtype group; NC, negative control, a colony bombarded with gold powders without plasmid DNA.

Following biolistic transformation and recovery for two days, all bombarded cells were transferred to liquid modified f/2 medium with 50 mg·L^−1^ of PPT and cultured for a week. During this selection process, most algal cells died and the color of medium turned from green to white. Then, the medium containing the herbicide was replaced with fresh modified f/2 medium for recovery. About one week later, the medium returned to green and approximately 10^5^ surviving cells were then transferred and spread on solid modified f/2 medium with 40 mg·L^−1^ of PPT. Thousands of colonies appeared after two weeks’ incubation (Figure 2a). Twenty-two colonies were selected and cultured in liquid modified f/2 medium without the addition of the herbicide for further molecular detection. No significant differences in growth or appearance could be observed between resistant and sensitive strains including blank and negative control (Figure 2b).

### 2.2. Detection of the bar and rt-PA Gene Integration

**Figure 3:**
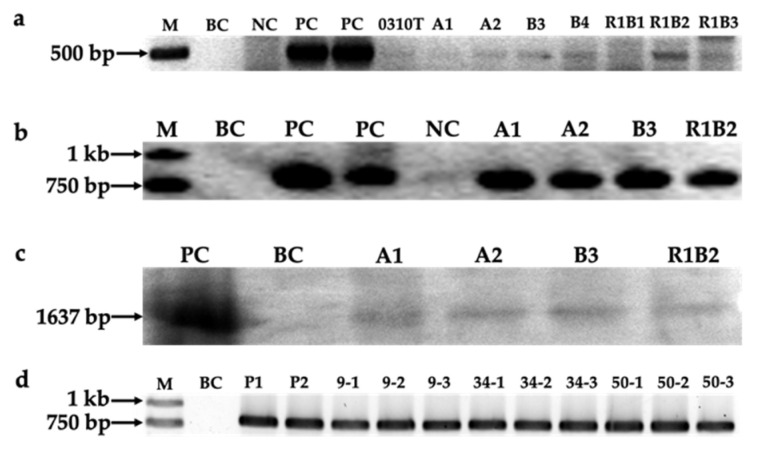
Results of PCR and Southern blotting detection for PPT resistance colonies. (**a**) PCR detection for *bar* gene; (**b**) PCR detection for *rt-PA* gene; (**c**) Southern blotting detection for colonies which had positive *rt-PA* gene signal in PCR detection; (**d**) PCR detection for *rt-PA* gene integration stability. M, marker; BC, blank control, the genomic DNA of wildtype algae strain; NC, negative control, the genomic DNA of algae strain bombarded with gold particles without plasmid DNA; PC, positive control, enzyme digestion product of plasmid vector pSVrPA/CaMVbar; P1, P2: B3 sample cultured with PPT; 9, 34, 50: B3 sample cultured without herbicide collected at day 9, 34 and 50.

Based on PCR detection results, eight (0310T, A1, A2, B3, B4, R1B1, R1B2, and R1B3) of the 22 colonies selected were detected to be positive with gene amplification of the 491 bp band of a *bar* gene fragment using primers bar1F/R (Figure 3a). The positive bands of integrated *bar* gene appeared faint mainly because the GC content of *bar* gene is high. Moreover, four colonies, A1, A2, B3, R1B2, showed the target 804 bp band amplified by primers rpa3F/R for a *rt-PA* gene fragment (Figure 3b). No target bands were detected in the wild-type sample or the negative control, which was transformed with naked golden particles. The four samples with both *bar* and *rt-PA* gene positive signals from PCR detection were analyzed by Southern blotting. The partial gene fragment of *rt-PA* gene and SV 40 poly(A) terminal signal amplified by primers rpa3F/R was used as the probe for Southern blotting. Total DNA of each sample was digested with endonucleases *Hin*dIII and *Bam*HI. An expected hybridization band was detected in all tested samples with no signal observed for the blank control (Figure 3c and Figure S1). The PCR and Southern blotting results showed that *rt-PA* was stably integrated into the genomes of colonies A1, A2, B3, and R1B2. To verify integration stability of *rt-PA* gene, B3 samples cultured for proliferation without herbicide PPT at day 9, 34, and 50 were collected. Another B3 sample maintained in f/2 medium with herbicide PPT for several months was also used. PCR detection using primers rpa3F/R showed that all samples contained *rt-PA* positive signals. (Figure 3d).

### 2.3. Purification of Recombinant Reteplase, Western Blotting and the Fibrinolysis Activity Assay

**Figure 4:**
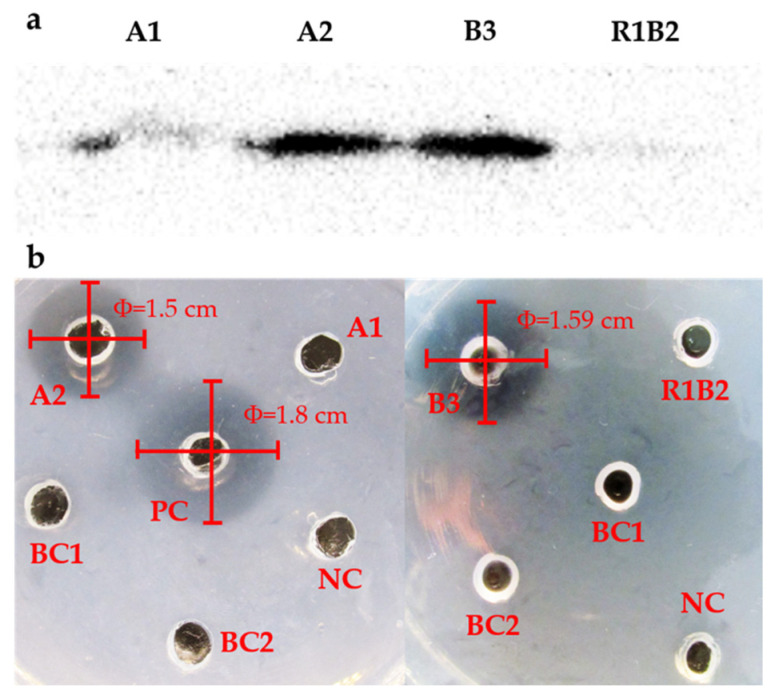
Results of Western blotting and FAPA detection for colonies A1, A2, B3, and R1B2. (**a**) Results of Western blotting detection; (**b**) FAPA experiment. BC1, blank control without any reagents in the well; BC2, blank control with PBS (used as protein solvent) in the well; NC, negative control with crude extract of soluble protein of *T. subcordiformis* in the well; PC, positive control with urokinase in the well.

The four colonies, A1, A2, B3, and R1B2, were cultured and proliferated in 2 L Erlenmeyer flasks to increase the biomass and for extraction of recombinant rt-PA protein. After the start codon ATG of *rt-PA*, a His-tag containing six histidine CAC codons was designed for affinity purification of recombinant rt-PA. The protein purification results showed that the recombinant protein was successfully transcribed and translated by colonies A1, A2, and B3, and rt-PA was produced as 1.91‰, 0.82‰, and 0.76‰ of the total soluble protein amount produced, respectively (Table 1), whereas no recombinant protein was produced by colony R1B2. The results of Western blotting followed by purification of Ni^2+^ affinity chromatography also demonstrated that recombinant rt-PA was produced by colonies A1, A2, and B3 (Figure 4a). The Western blot of A1 was fainter than that of A2 and B3, probably because a portion of proteins expressed in A1 were folded incorrectly and the N-terminal domain was incorporated into the inside of the protein, which resulted in a change of protein secondary construction, and the reaction between antigen and antibody failed in Western blotting. The bioactivity of recombinant rt-PA was verified using the fibrin agarose plate assay (FAPA) method. FAPA provides a quick, sensitive method to examine fibrinolysis activity. rt-PA converts plasminogen into plasmin, which then degrades fibrin to produce cleared zones. According to the results of FAPA, the transparent circle observed for colonies A2 and B3 showed effective bioactivity of rt-PA produced by *T. subcordiformis*, and no such activity was observed in blank and negative controls (Figure 4b).

## 3. Discussion

The results showed that *rt-PA* was successfully integrated into the genome of *T*. *subcordiformis*, and recombinant rt-PA expressed by colonies A2 and B3 showed expected thrombolytic bioactivity, indicating that recombinant rt-PA underwent proper post-translational modification and structural folding in marine microalga *T*. *subcordiformis*.

Although a stable nuclear genetic transformation system has been established in *T*. *subcordiformis* [10], rigorous screening and detection procedures are essential for obtaining positive transformants that stably express non-native proteins with bioactivity. The bombarded cells were first screened in liquid medium against PPT to remove the majority of untransformed cells. The change in the color of the medium from green to white demonstrated that most cells were killed by this screening step. Subsequently, the second screening on a solid agar plate was performed to obtain a monoclonal algal strain. In this step, 10^5^ cells were spread on the solid medium plate and thousands of colonies appeared. Twenty-two colonies were selected from the plate for PCR detection, and only eight colonies yielded a positive *bar* gene signal, from which only four colonies were found to be positive for the *rt-PA* gene. These results indicated that the majority of the 22 clones grown on the agar selection plate were herbicide tolerant rather than resistant clones, and co-integration of *bar* with *rt-PA* was not a dominant event. Additionally, Western blotting analysis identified a clone, R1B2, without the expected protein product, suggesting potential non-integration of expression regulatory elements, or gene silencing from a position effect. Finally, only two of the three clones, A2 and B3, were confirmed to express recombinant rt-PA with thrombolytic activity by FAPA, indicating that incorrect post-translational modifications may have occurred. The products produced by A1 resulted in loss of bioactivity. Therefore, multiple rounds of screening and detection assays are necessary to achieve engineered microalgal strains with expected bioactivity.

In this study, the average concentration of rt-PA expressed in strains A1, A2, and B3 was 1.16 μg·mg^−1^ soluble protein, which is approximately seven-fold higher than that expressed in gametophytes of the brown macroalga *Saccharina japonica*, in which 0.159 μg·mg^–1^ soluble protein was produced [19]. Presumably, this is because the protein content of *S. japonica* was much lower than that of *T*. *subcordiformis*. In these three rt-PA protein positive strains, A1 exhibited the highest concentration of 1.91 μg·mg^−1^. However, thrombolytic bioactivity was not detected in this strain. The integrated *rt*-*PA* gene of A1 was sequenced and the coding region did not acquire any mutations. According to results of SDS-PAGE (Figure S2) and Western blot, the recombinant rt-PA was indeed translated in colony A1 and the size of the recombinant protein was correct. Based on the above results, it was suggested that the primary structure of recombinant rt-PA has no mistake. The bioactivity of rt-PA also relies heavily on the secondary structure, particularly the accurate cleavage on the specific site and the correct form of the nine disulfide bond. We inferred that the secondary structure of recombinant rt-PA was not correct in A1. Verification of this hypothesis needs more experimental evidence. Currently, the main preparation method of Reteplase is recombinant expression in *Escherichia coli*. The recombinant rt-PA prepared using this recombinant expression system is usually produced in inclusion bodies as an inactive form that is only active after in vitro folding [20,21]. The renaturation ratio is relatively low, which contributes to the associated high costs of preparing this drug. Our research showed that using photoautotrophic green microalga, a eukaryotic expression host, to produce bioactive Reteplase is feasible. The recombinant expression product of A2 and A3 strains was bioactive, indicating that produced recombinant rt-PA underwent proper post-translational modification and structural folding in *T*. *subcordiformis*.

In addition to using this new marine bio-reactor to produce recombinant drugs, the successful expression of recombinant proteins with complex structures also makes it possible to use microalga to deliver drugs or vaccines though the food chain. *T*. *subcordiformis* is a common green microalga living in offshore areas of China. The shape of the cell is compressed and generally 11–16 µm in length, 7–9 µm in width, and 3.5–5 µm in thickness [22]. In addition, *T*. *subcordiformis* is enriched with polysaccharides, proteins, fatty acids, and vitamins [4,5,6,7]. In view of the above characteristics, this microalga is an easy and suitable prey for larvae of fish, shrimp, and shellfish, and the technology for large-scale cultivation has been established [23]. Currently, antibiotic abuse is a prominent problem in aquaculture [24]. Among the possible solutions to resolve this issue, oral administration of microalgae that contain recombinant antibacterial peptides or vaccines is a promising solution [25].

In conclusion, using the established stable nuclear transformation system, Reteplase with a complex secondary structure encoded by *rt-PA* was expressed successfully in the marine green microalga *T. subcordiformis*. This research demonstrated that *T. subcordiformis* has potential use as a promising cell factory to prepare bioactive proteins such as recombinant drugs and other substances. To elevate the expression level of Reteplase in *T. subcordiformis*, more efforts are needed in searching for endogenous stronger promoters, screening more positive transformants, and optimizing conditions of growth and fermentation.

## 4. Materials and Methods

### 4.1. Algae Culture

The strain of *T. subcordiformis* was provided by Professor Song Xue from Dalian Institute of Chemical Physics, Chinese Academy of Sciences. *T. subcordiformis* cells were cultured in a modified f/2 medium [26] at 23 °C with a 12:12 h light:darkness photoperiod under 90–100 µmol photons m^–2^·s^–1^ irradiance. The culture density was measured using a hemocytometer and cells for genetic transformation were harvested at the mid-log phase.

### 4.2. Plasmid Vector for Transformation

The heterologous protein expression vector pSVrPA/CaMVbar constructed in our laboratory was used [19]. The vector was maintained and amplified in *E. coli* Top10 strain. Plasmid DNA was isolated from *E. coli* with a TIANpure Midi Plasmid Kit (Tiangen, Beijing, China).

### 4.3. Biolistic Transformation

Genetic transformation was performed using a Biolistic PDS-1000/He Particle Delivery System (Bio-Rad, Hercules, CA, USA). Before bombardment, *T. subcordiformis* at log phase were collected by centrifugation at 6000 rpm (approximately 3500× *g*) for 5 min. Approximately 1 × 10^8^ cells were then spread onto the central area of f/2 medium agar plates to a diameter of 2 cm. According to the operation protocol described by Cui et al. [12], the transformation vector was adhered onto gold particles (1.0 µm in diameter), and the same parameters described in this protocol were followed, including the 900 psi of rupture disk pressure and a particle travel distance of 6 cm. After transformation, plates were placed in darkness for 8 h for recovery. A plate bombarded with gold powder without plasmid DNA was used as a negative control. All experiments were performed in triplicate.

### 4.4. Basta Selection for Positive Transformants

After two days of culture, transformed cells were transferred to the selective liquid modified f/2 medium with 60 mg·L^–1^ of Basta and cultured for a week. Surviving cells were then spread on solid modified f/2 medium agar plates with 40 mg·L^–1^ of Basta. Colonies appearing after two weeks were selected and streaked on agar plates of modified f/2 medium with 20 mg·L^–1^ Basta. After another two weeks of cultivation, 22 colonies were selected and cultured in the liquid modified f/2 medium without the addition of the herbicide.

### 4.5. PCR detection and Southern Blotting

After two weeks of cultivation in liquid medium without the selection pressure of the herbicide, cells originating from selected resistant colonies were harvested and genomic DNA was extracted using a plant genomic DNA kit (Tiangen, Beijing, China). According to the sequence of vector pSVrPA/CaMVbar, primers bar1F (5′-TCTGCACCATCGTCAACCACTACA-3′), bar1R (5′-TCAAATCTCGGTGACGGGCAGGAC-3′), rpa3F (5′-TCTTGGGCAGAACATACC-3′), and rpa3R (5′-TCCCCCTGAACCTGAAAC-3′) were designed to amplify the specific *bar* and *rt-PA* gene fragments by PCR. PCR products were visualized using gel electrophoresis in a 1.2% agarose gel stained with Super GelRed (US Everbright Inc., Suzhou, China). The probe of *rt-PA* gene for Southern blotting was synthesized using the 804 bp PCR fragment amplified by primers rpa3F/R. Total genomic DNA (about 10 µg) of each sample was digested with endonucleases *Hin*dIII and *Bam*HI (New England Biolabs, Ipswich, MA, USA). Probe synthesis and Southern blotting were conducted with a DIG DNA labeling and detection kit (Roche, Mannheim, Germany).

### 4.6. Purification of Recombinant Reteplase and Western Blotting

Positive transformants transformed with pSVrPA/CaMVbar were frozen in liquid nitrogen and then homogenized using a plant tissue grinder Tissuelyser-48 (Jingxin, Shanghai, China) with a vibration frequency of 70 Hz. The duration time was 90 s and the homogenizing process was repeated three times. Then, 200 µL extraction buffer consisting of 10 mM Tris (pH 8.0), 0.02% NaN_3_, and 0.001% PMSF was added to 1.5 mL microcentrifuge tubes with homogenates from 100 mg fresh thalli. The mixture was centrifuged at 12,000× *g* for 15 min at 4 °C and the supernatants were transferred to new microcentrifuge tubes and stored on ice for further use. The total soluble protein concentration was determined by using the Bradford Protein Assay Kit (Tiangen, Beijing, China).

To purify expressed rt-PA, the prepared supernatants were mixed with 30 mL binding buffer (20 mM NaPO_4_, 500 mM NaCl, and 20 mM imidazole, pH 7.4), and loaded onto a pre-equilibrated column of Chelating Sepharose (GE Healthcare BioScience, Marlborough, MA, USA) charged with Ni^2+^. The column was washed with washing buffer (20 mM NaPO_4_, 500 mM NaCl, and 100 mM imidazole, pH 7.4) to remove miscellaneous proteins. rt-PA was eluted with elution buffer (20 mM NaPO_4_, 500 mM NaCl, and 500 mM imidazole, pH 7.4). The imidazole was removed by using a Sephadex G25 column. The peak fraction was collected and concentrated by ultrafiltration.

The expression of recombinant protein was analyzed by 12% sodium dodecyl sulfate-polyacrylamide gel electrophoresis and Western blotting. After electrophoresis, the proteins were transferred onto a polyvinylidene fluoride membrane using the Trans-Blot^®^ SD semi-dry blotting system (Bio-Rad), with a constant voltage of 15 V for 60 min at room temperature. The blotted membrane was then blocked in 5% skimmed milk powder solution for 60 min. Subsequently, the membrane was incubated in a solution containing HRP Conjugated Anti His-Tag Mouse Monoclonal Antibody (CWBIO, Beijing, China) for 2 h at room temperature, and finally was dyed with HRP-DAB agent (Tiangen, Beijing, China) for 5 min.

### 4.7. Fibrinolysis Activity Assay

The bioactivity of purified rt-PA was measured using a modified fibrin agarose plate assay (FAPA) method [19]. The prepared fibrin agarose plate was prepared with 1.0% agarose, 1 mg·mL^–1^ bovine fibrinogen, and 0.1 U·mL^–1^ bovine thrombin in PBS (pH 7.4). Holes with diameters of 2–4 mm were made in the plate using an Oxford cup. In each hole, 20 µL PBS (pH 7.4) containing 0.04 U bovine plasminogen and urokinase or purified protein was added and followed by incubation for 16 h at 37 °C. Bovine fibrinogen, thrombin, plasminogen, and urokinase were obtained from the National Institute for the Control of Pharmaceutical and Biological Products (NICPBP, Beijing, China). Two blank controls were used in the FAPA experiment: an empty well without any reagents (BC1) and a well filled with PBS (used as protein buffer) (BC2). The negative control was crude soluble protein extract from *T. subcordiformis*. Urokinase was used as a positive control.

## Figures and Tables

**Table 1:** Quantitative determination of recombinant rt-PA in transgenic samples.

Samples	Biomass (Fresh Weight)	Soluble Protein	rt-PA	Concentration (μg·mg^−1^ Soluble Proteins)
A1	1.93 g	23.2 mg	44.2 μg	1.91
A2	2.20 g	24.8 mg	20.4 μg	0.82
B3	2.34 g	23.6 mg	17.85 μg	0.76
R1B2	1.65 g	23.6 mg	-	-

## Data Availability

The data presented in this study are available within this article and supplementary materials.

## Supplementary Materials

The following are available online at https://www.mdpi.com/article/10.3390/md19060315/s1, Figure S1. Southern blotting detection for colonies A1, A2, B3 and R1B2. Figure S2. The SDS-PAGE gel of colony A1, A2 and B3.
