# Supplementary material for: Recombinant Expression of Thrombolytic Agent Reteplase in Marine Microalga Tetraselmis subcordiformis (Chlorodendrales, Chlorophyta)
Source: Mar Drugs. 2021 May 28;19(6):315. doi: 10.3390/md19060315 (PMC8230124; doi:10.3390/md19060315)
Supplement: Supplementary file 1 [file marinedrugs-19-00315-s001.zip › marinedrugs-1186902-supplementary.pdf]

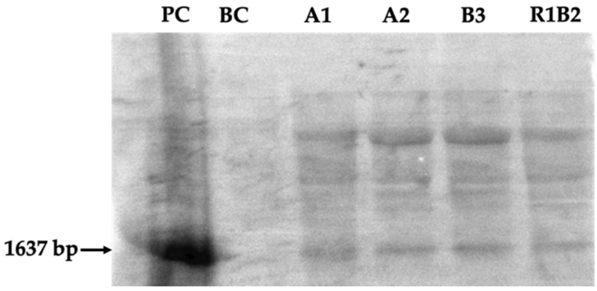

Figure S1. Southern blotting detection for colonies A1, A2, B3 and R1B2.

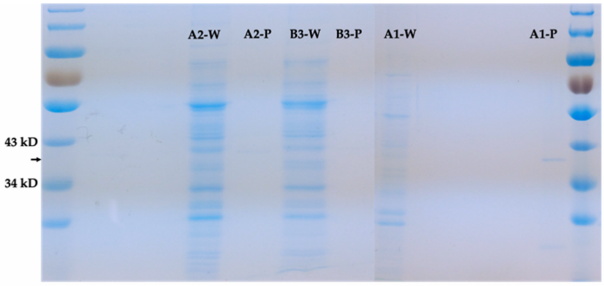

Figure S2. The SDS-PAGE gel of colony A1, A2 and B3. W: total soluble proteins extracted from *T. subcordiformis*; P: purified proteins with Ni^{2+}-NTA affinity chromatography.
